# State of the art of robotic pancreatoduodenectomy

**DOI:** 10.1007/s13304-021-01058-8

**Published:** 2021-05-20

**Authors:** Niccolò Napoli, Emanuele F. Kauffmann, Fabio Vistoli, Gabriella Amorese, Ugo Boggi

**Affiliations:** 1 Division of General and Transplant Surgery, University of Pisa, Pisa, Italy; 2 Division of Anesthesia and Intensive Care, Azienda Ospedaliera Universitaria Pisana, Pisa, Italy

## Abstract

Current evidence shows that robotic pancreatoduodenectomy (RPD) is feasible with a safety profile equivalent to either open pancreatoduodenectomy (OPD) or laparoscopic pancreatoduodenectomy (LPD). However, major intraoperative bleeding can occur and emergency conversion to OPD may be required. RPD reduces the risk of emergency conversion when compared to LPD. The learning curve of RPD ranges from 20 to 40 procedures, but proficiency is reached only after 250 operations. Once proficiency is achieved, the results of RPD may be superior to those of OPD. As for now, RPD is at least equivalent to OPD and LPD with respect to incidence and severity of POPF, incidence and severity of post-operative complications, and post-operative mortality. A minimal annual number of 20 procedures per center is recommended. In pancreatic cancer (versus OPD), RPD is associated with similar rates of R0 resections, but higher number of examined lymph nodes, lower blood loss, and lower need of blood transfusions. Multivariable analysis shows that RPD could improve patient survival. Data from selected centers show that vein resection and reconstruction is feasible during RPD, but at the price of high conversion rates and frequent use of small tangential resections. The true Achilles heel of RPD is higher operative costs that limit wider implementation of the procedure and accumulation of a large experience at most single centers. In conclusion, when proficiency is achieved, RPD may be superior to OPD with respect to CR-POPF and oncologic outcomes. Achievement of proficiency requires commitment, dedication, and truly high volumes.

## Introduction

First performed by Codivilla in 1898 [1], pancreatoduodenectomy (PD) is commonly known as “Whipple procedure”, in honor of Allen Oldfather Whipple who reported the first successful one-stage PD in 1941 [2]. After 80 years of refinements, PD remains associated with high morbidity and mortality rates. A study published in the New England Journal of Medicine in 2011 showed that risk-adjusted mortality of all types of pancreatic resection in 2008 (5.5%) was only slightly inferior to that of aortic valve replacement (6.6%) and superior to that of coronary artery bypass (3.4%) [3]. Considering that these figures refer to open pancreatic surgery, it is clear that open surgery offers opportunity for improvement.

In recent years, minimally invasive surgery has improved the outcome of many abdominal operations. Feasibility of minimally invasive PD was shown over 25 years ago [4], but the procedure did not gain widespread popularity due to the intrinsic technical limitations of conventional laparoscopy, the need to overcome a steep learning curve, and the lack of clear clinical benefits. A worldwide survey on opinions and use of minimally invasive pancreatic resections demonstrated that 29% of responding surgeons performed minimally invasive PD. The most common reasons for not performing minimally invasive PD were lack of specific training (62%), difficulty of surgical technique (44%) and lack of time in surgical schedule (37%). Interestingly, while current value of minimally invasive PD was deemed superior to that of open PD (OPD) only by 17% and 7% of surgeons performing and not performing minimally invasive PD, respectively, equivalent figures for future value were 53% and 23%, respectively [5].

The da Vinci Surgical System® (Intuitive Surgical, Sunnyvale, CA, USA) enhances surgical dexterity in minimally invasive procedures and, therefore, provides the opportunity to verify if a minimally invasive approach can improve the outcome of PD. We herein present the state of the art of robotic PD (RPD).

## Historical and technical notes

The first RPD was performed in 2001. This landmark procedure was reported by Giulianotti and coworkers in 2003 in an article presenting the use of the dVSS in general surgery [6]. In the first 6 RPDs, dissection was carried out laparoscopically and robotic assistance was used only for digestive reconstruction. In the remaining two patients, a full robotic technique was adopted. A hybrid approach to RPD is still very popular, and is used even at major centers [7], mostly because of the “rigidity” of the dVSS and the lack of articulated streamlined energy devices suitable for retroperitoneal dissection. A fully robotic procedure, however, is also possible [8, 9]. Currently, there is no agreed technical standard for RPD. Evidence-based guidelines for minimally pancreatic resections recommend a minimum annual volume of 20 procedures per center without distinction between RPD and LPD [10]. There is no evidence on minimum volume requirements for individual surgeons.

## Feasibility and learning curve

Feasibility of RPD has been demonstrated by several independent groups [9, 11–13]. A collaborative study reporting on the outcome of the first RPDs performed at 5 centers between January 2008 and August 2014 demonstrated that RPD can be safely implemented in selected patients at high-volume centers. In detail, in a total of 92 patients with a mean age of 65 ± 12 years, a mean body mass index of 25.8 ± 5.0 kg/m^2^, a prevalence of male gender (53%), and a proportion of ASA III patients of 46%, median operating time was 504 min (interquartile range 133), median estimated blood loss was 242 ml (interquartile range 398), and conversion to open surgery was required in 12 procedures (13%). Regarding pancreatic anastomosis, pancreaticojejunostomy was employed in all but 2 patients and temporary ducts stents were inserted in the majority of patients (69.5% overall, and 86.7% in patients with pancreatic duct diameter < 3 mm) irrespective of type of anastomosis (i.e., invaginating or duct-to-mucosa). Clinically relevant post-operative pancreatic fistula (CR-POPF) developed in 9 patients (9.9%; 4 grade B and 5 grade C). Rate of severe post-operative complications was 24% with 2 (2.2%) deaths and ten (10.9%) reoperations. A margin negative resection was achieved in 75% of the patients with pancreatic cancer with mean harvest of 16 ± 8 lymph nodes [14]. In subsequent studies, RPD was associated with extremely low rates of conversion to open surgery (ranging from 1.1% to 5.1%) [15, 16]. When compared with OPD, RPD required longer operating times [17], but reduced blood loss and need for blood transfusions [18]. In an early study, Chalikonda and coworkers reported a post-operative death caused by a portal vein injury requiring emergent conversion to open [12]. This dreadful occurrence is not specific to RPD and has already been reported also for laparoscopic PD (LPD) [19]. In a recent collaborative study, conversion to open was required in 65 of 709 minimally invasive PDs (9.1%), including 48 elective conversions (6.7%) and 12 emergency conversions (1.6%). Reasons for conversion were unknown in 5 patients. The incidence of conversion in LPD was twice as high when compared to RPD [52 of 459 (11.3%) versus 13 of 250 (5.2%); *p* = 0.007]. At multivariable analysis, using RPD as reference, LPD was strongly associated with conversion to open surgery (OR 5.2; 2.5–10.7; *p* < 0.001). Conversions were more frequent in medium-volume centers (10–19 procedures per year) than in high-volume centers (15.2% versus 4.1%; *p* = 0.001) [20].

Several studies have described the learning curve for RPD, mostly using cumulative summative (CUSUM) analysis of operating time. Excluding one article defining the simultaneous learning curve of two surgeons at 80 procedures [21], the number of cases required to overcome the learning curve for a single surgeon ranged between 20 and 40 RPDs [22–26]. Implementation of mentorship and a proficiency-based curriculum could not affect POPF rate, but was shown to reduce operating times, conversion rates, severe post-operative complications, and estimated blood loss [27]. A recent systematic review on the learning curve of LPD and RPD showed that the learning curve of a single surgeon was considerably longer for LPD [49.8 (95% CI 43.8–56.4) versus 28.3 (23.3–34.0); IRR: 1.76, 95% CI 1.04–2.99; *p* = 0.0360], while the Institutional learning curve was longer for RPD [43.6 (95% CI 38.0–49.8) versus 21.0 (95% CI 17.5–25.0)] although the difference was not statistically significant [28].

Two recent studies from extremely high-volume centers showed that true completion of the learning curve may require as many as 250 procedures [16, 17]. After this, the number of RPDs outcomes was optimized [29].

One study showed that based on operating times, 35 cases are required to overcome the learning curve of RPD with vein resection. Completion of the learning curve, however, was associated only with reduction in length of hospital stay, without improvement in estimated blood loss, margin status, post-operative pancreatic fistula, severe complications, and post-operative mortality [30].

## Post-operative pancreatic fistula

Few studies, all reporting retrospective analyses, have compared the outcomes of LPD and RPD [31–35]. Most of these studies have not shown an advantage of RPD in terms of occurrence of CR-POPF. However, a large multicenter study showed that single-row pancreatojejunostomy when performed during minimally invasive PD increased the incidence of POPF (OR 2.95, P < 0.001) and that this type of anastomosis was prevalent in LPD [36]. An additional study confirmed that the use of single-row pancreatojejunostomy in minimally invasive PD was prevalent in LPD (16%), as compared to either RPD (13%) or hybrid PD (1%), and that it was independently associated with the development of CR-POPF (OR 5.0, 95 CI 3.0–8.2). The authors of this study speculate that inferiority of laparoscopic single-row pancreatojejunostomy could be caused by the increased technical difficulty experienced in LPD, as inferiority single-row anastomosis was not seen in either RPD or OPD [37].

A recent study by Shi and coworkers reported on the outcomes of 200 RPDs performed after the first 250 cases. In this study, RPD was found to be superior to OPD for several parameters, but not for CR-POPF. However, in matched cohorts, CR-POPF occurred less frequently after RPD (10.2% versus 14.4%). The difference, although not statistically significant, was well evident for grade C POPF (3.7% versus 7.5%). RPD improved operating time, estimated blood loss, and length of hospital stay [29].

Cai and coworkers showed a lower incidence of CR-POPF after RPD when compared to OPD (6.7% versus 15.8%, *p* < 0.001). In detail, grade B POPF occurred more frequently after OPD (13.3% versus 2.5%, *p* < 0.001), while the incidence of grade C POPF was similar in the two groups (2.5% vs. 2.0%, *p* = 0.470). RPD was protective against CR-POPF in case moderate-risk anastomoses (7.1% vs. 15.2%, *p* = 0.008). Lower rates of CR-POPF were also observed in in low- and high-risk anastomoses, although difference did not reach statistical significance. RPD remained an independent predictor of lower CR-POPF on multivariate analysis (OR 0.278, *p* < 0.001) and continued to be protective after propensity matching (coefficient =  − 0.113, *p* = 0.001) [38].

Lower rates of CR-POPF in RPD versus OPD (11.9% vs 15.6%; *p* = 0.026) were shown also in a retrospective analysis of the American College of Surgeons National Surgical Quality Improvement Program (ACS-NSQIP) database. In this study, RPD was also associated with decreased median time to drain removal (4 vs 7 days; *p* < 0.001). Despite a similar incidence of CR-POPF in RPD and LPD, more patients required a percutaneous catheter drainage after LPD (10.8% vs 15.7%; *p* = 0.030) [39].

## Other post-operative complications

In a study by Vining and coworkers, RPD (versus OPD) was associated with shorter median length of stay (7 vs 8 days; *p* < 0.001), and lower rates of any complication (46.8% vs 53.3; *p* = 0.004), surgical complications (42.6% vs 48.6%; *p* = 0.008), hemorrhage requiring blood transfusions, (10.4% vs 17.7%; *p* < 0.001), wound complications (6.2% vs 9.1%; p = 0.029), wound dehiscence (0.2% vs 1.3%; *p* = 0.035), sepsis (6.2% vs 9.3%; *p* = 0.019) and pneumonia (1.6% vs 3.8%; *p* = 0.012). In comparison with LPD, RPD was associated with lower rates of bleeding requiring transfusions (10.4% vs 17.4%; *p* = 0.002). Rates of 30-day readmission were higher after RPD than after either OPD (24.3% vs 16.2%; *p* < 0.001) or LPD (24.3% vs 15.5%; *p* = 0.001) [39].

In a study by Shi and coworkers, RPD (versus OPD) was associated with reduced mean blood loss (297.3 ± 246.8 versus 415.2 ± 497.9 mL; 95% CI  − 197.8848 to − 38.0510; *p* = 0.002), lower rate of intra-abdominal infections (21.4% versus 34.2%; *p* = 0.008) and shorter duration of hospital stay (22.4 ± 16.7 versus 26.1 ± 16.3] days; 95% CI  − 7.0837 to − 0.3708; *p* = 0.03). No difference was noted in bile leak, delayed gastric emptying, post-operative bleeding and need for reoperation [29].

Aguayo and coworkers in a study aiming to investigate readmission rates in RPD and OPD, based on the National Readmission Database (January 2010 to December 2017), showed that out of 81,457 patients who survived the index hospitalization (96.9%), 15,823 (19.4%) required 30-day hospital readmission (RPD: 19.5% versus OPD: 18.6%; *p* = 0.34). RPD was associated with reduced length of index hospital stay (12.3 days versus 14.0 days; *p* = 0.002). Incidence of complications at index hospitalization was similar between RPD and OPD (27.7% versus 25.5%: *p* = 0.28), with lower rates of Clostridium difficile infections in OPD (2.6% versus 1.3%; *p* = 0.03), and a trend for fewer intraoperative complications in RPD (0.9% versus 4.8%; *p* = 0.06) [40].

Compared to LPD, RPD was associated with reduced operating time [32], lower estimated blood loss [32], fewer conversions to open surgery [32–35] and shorter hospital stay [32].

## Postoperative mortality

Data from the literature show that RPD does not increase post-operative mortality when compared to either OPD [29, 35, 40] or LPD [33, 39, 41].

## Adoption

Adoption of surgical innovation typically follows an S-shaped curve and occurs in five stages. Adoption accelerates to a peak when innovation enters the third stage and innovation is accepted by the “early majority” [42]. This typically occurs when safety is shown and efficacy starts to become evident.

Two studies have shown a recent increase in the use of robotic assistance for pancreatic operations. An analysis of the National Inpatient Sample database (2010–2014) showed that there was a fivefold general increase in the use robotic surgery (compared to 1.1-fold increase in laparoscopy and 1.2-fold decrease in open surgery). The use of robotic assistance for pancreatic resections increased from < 1% in 2010 to 3% in 2014. When compared with laparoscopy, the odds having of robotic pancreatic surgery increased from 3.95 (0.85–18.24) in 2011 to 7.94 (3.53–17.85) in 2014 [43]. An additional study analyzing the National Cancer Database (2010–2014) demonstrated increased use of robotic assistance for pancreatic resections, with a decrease in post-operative morality (from 6.7 to 1.8%) and an increase in the number of examined lymph nodes (from 18 to 21) for RPD [44].

## RPD for pancreatic cancer

Girgis and coworkers reported a retrospective analysis of 456 patients who received either a robotic (226) or open (230) pancreatectomy for pancreatic cancer (PD: 361; distal pancreatectomy: 73; distal pancreatectomy with celiac axis resection: 22). In PD the robotic approach (versus OPD) was associated with similar rates of R0 resection, but higher lymph node harvest (21.47% versus 21.72%), (31.9 ± 12.2 versus 25.9 ± 11.1; *p* < 0.0001), lower estimated blood loss [250 mL (150, 400) versus 500 mL (300–925)], and lower need of blood transfusions (17.2% versus 40.4%; *p* < 0.0001). A similar proportion of patients in the two groups received neoadjuvant chemotherapy (61.11% versus 61.93%), adjuvant chemotherapy (67.90% versus 71.35%), and both neoadjuvant and adjuvant chemotherapy (41.6% versus 43.5%). RPD was associated with lower rates of associated vein resection (25.2% versus 37.9%; *p* = 0.01) and higher rates of administration of < 6 cycles of adjuvant chemotherapy (46.6% versus 36.1%; *p* = 0.047). Time to adjuvant chemotherapy was slightly longer in RPD [65 days (56–81) versus 62 days (48–83); *p* = 0.056]. On multivariable analysis, RPD (HR 0.75, *p* = 0.05), severe complications ((HR 1.45, *p* = 0.006), presence of lymph node metastasis (HR 1.62; *p* = 0.003) and R1 resection (HR 1.55; *p* = 0.001) predicted survival [45].

Nassour and coworkers in an analysis of the National Cancer Database (2010–2016) identified 626 RPD and 17,205 OPD performed for stage I–III pancreatic cancer. RPD was associated with higher mean number of examined lymph nodes (12 vs 11; *p* < 0.001) and procedures with > 12 lymph nodes (81% versus 73%; *p* < 0.001). There was no difference in median overall survival between RPD (22.0 months) and OPD (21.8 months). RPD was not associated with inferior overall survival [hazard ratio (HR) = 1.014; 95% CI 0.903–1.139] [46].

In an additional study, Nassour and coworkers queried the National Cancer Database (2010–2013) for RPD (*n* = 165) and LPD (*n* = 1458). Most procedures in both groups were performed for pancreatic cancer (89.1% versus 90.1). RPD was associated with higher mean number of harvested lymph nodes (19.3 ± 18.0) vs 17.2 ± 17.0) *p* = 0.081) and lover conversion rate (17.0% versus 27.6%; *p* = 0.003). No difference was found in median overall survival (20.7 months versus 22.7 months; *p* = 0.445) [34].

Little evidence is available to define feasibility and safety of RPD in patients receiving neoadjuvant treatments that are currently used in patients with borderline-resectable pancreatic cancer and in some patients with anatomically resectable tumors.

## RPD with vascular resection

Approximately 30% of the patients with a cancer located in the pancreatic head are classified borderline resectable and may require a vein resection.

Vein resection during RPD was first reported Giulianotti and coworkers in 2 patients. The first patient received a stapled type 1 resection. The second patient had a type 2 resection using a polytetrafluoroethylene patch [47]. Our group was the first to report arterial resections in 4 RPDs [48].

Beane and coworkers reported a retrospective review of 380 consecutive RPDs (October 2011–May 2017) including 50 patients with associated vein resection and reconstruction. The majority of patients (*n* = 43; 86.0%) received a type 1 resection using a vascular stapler. The remaining 7 patients received either a type 2 resection (using a bovine pericardial patch for repair) or a type 3 resection. Mean operating time was 412 ± 82 min (412 ± 82 min in type1 resections and 463 ± 109 min in type 2–3 resections). Conversion to open surgery was required in 8 of 43 type 1 resections (18.6%) and in 3 of 7 (42.8%) type 2–3 resections. Mean (range) estimated blood loss was 250 mL (200–500 mL) in type 1 resections, and 400 mL (200–1500 mL) in type 2–3 resections. RPD with vascular resection was associated with a median length of hospital stay of 7 days (6–9), an incidence of severe complications of 28.0%, a 90-day readmission rate of 43.0%, a 30-day mortality of 4.0% and a 90-day mortality of 8.0% [30].

Shyr and coworkers reported a retrospective review of the experience of the Taipei Veterans General Hospital (2012–2018). A vein resection was required in 43 of 391 PDs (10.9%), including 32/208 (15.3%) OPDs and 11/183(6.0%) RPDs (*p* = 0.003). Conversion to open surgery was required in 36.4% of RPDs with vein resection [49].

Marino and coworkers reported a retrospective review of 83 RPDs (March 2013–October 2019), including 10 procedures with vein resection (12.0%). When compared to standard RPD, RPD with vascular resection was associated to longer mean operating time (642 ± 105.7 min versus 525 ± 92.3 min; *p* = 0.003), higher median estimated blood loss [620 mL (380–1100.5) versus 290 mL (155–494.5); *p* = 0.002], and more frequent use of blood transfusions (20.0% versus 5.5%; *p* = 0.002). No difference was noted in rate of conversion to open surgery (10.0% versus 6.8%), reoperation (20.0% versus 8.2%), median length of hospital stay [13 days (6–19) versus 10 days (5–19)], 90-day readmission (10.0% versus 6.8%), and 90-day mortality (10.0% versus 4.1%). In patients with pancreatic cancer (6 versus 38), RPD with vein resection was associated with higher mean number of examined lymph nodes (46 ± 7 versus 32 ± 11; *p* = 0.03) and equivalent rates of R0 resection (83.3% versus 92.1%), local recurrence (16.7% versus 13.2%), 1-year overall survival (85.7% versus 81.6%), and 1-year disease-free survival (71.4% versus 71.1%) [50].

A retrospective analysis of our experience revealed that between October 2008 and April 2016 14 patients had RPD with vein resection out of total of 130 RPDs (10.7%). No patients undergoing RPD with vein resection required conversion to open surgery. Type 2 vein resection was performed in one patient (7.1%), type 3 in 5 patients (35.7%), and type 4 in 8 patients (57.2%). Compared to OPD, RPD with vein resection was associated with longer mean operating time (640.7 ± 99.7 min versus 521.7 ± 98.7 min; *p* = 0.0006), higher median estimated blood loss [1109.7 mL (819.4–1430.2) versus 419.5 mL (177.5–689.6); *p* < 0.0001], more frequent use of intraoperative blood transfusions (28.6% versus 3.5%; 0.0047), and more frequent diagnosis of pancreatic cancer (57.1% versus 19.8%; *p* = 0.002). No differences were noted in incidence of severe post-operative complications (28.6% versus 17.2%) median length of hospital stay [21.5 days (15–33.3) versus 18 days (14–25.8)], 90-day readmission (0 versus 8.8%), number of examined lymph nodes (60 ± 13.9 versus 57.2 ± 14.6), rate of R0 resections (75.0% versus 83.3%). A trend to higher 90-day post-operative mortality was noted in the RPD with vein resection (14.3% versus 1.7%; *p* = 0.06), but cases were not matched for baseline characteristics that could predict survival [51].

## COSTS

In a study on feasibility, we showed that RPD, when compared to OPD, is associated with a total amount of added costs of 6193 Euro [10]. Rosemurgy and coworkers in an economic analysis showed that total costs of care was 31,389 US dollars (36,611 ± 20,545.4) for RPD and 23,132 US dollars (31,323 ± 28,885.5) (*p* = 0.04) for OPD [52]. On the contrary, Aguayo and coworkers showed that index costs were not significantly different between RPD and OPD ($51,956 versus $47,296, *p* = 0.28) [40].

## Discussion

Available data suggest that robotic assistance improves the outcomes of minimally invasive PD. In general, RPD is as safe ad OPD [35, 40] and LPD [33, 39, 41]. Compared to LPD, RPD reduces the overall risk of conversion to open surgery and the risk of emergency conversion due to major bleeding [20], thus addressing one of the major concerns on safety of minimally invasive PD [19]. Under appropriate conditions, RPD facilitates safe implementation of minimally invasive PD [14] and reduces the learning curve of this procedure, when compared to LPD [28]. Availability of mentorship and implementation a proficiency-based curriculum further facilitate safe implementation of RPD [27].

Despite current excellent data, the full potential of RPD could not have been fully explored yet. Indeed, two recent studies demonstrate that after 250 procedures outcomes of RPD are optimized [15, 16] and become superior to those of OPD [29]. Considering that also in OPD excellence is reached only with high institutional [53] and individual [54] volumes, RPD promises to show clear superiority over alternative approaches.

As for now, RPD is at least equivalent to OPD and LPD with respect to incidence and severity of POPF, incidence and severity of post-operative complications, and post-operative mortality [31–35]. In patients diagnosed with pancreatic cancer, RPD is associated with similar rates of R0 resections, but higher number of examined lymph nodes, lower blood loss, and lower need of blood transfusions [34, 45, 46]. Multivariate analysis shows that RPD could improve patient survival [45]. Limited evidence shows that RPD with vein resection and reconstruction is feasible [30, 49–51], but at the price of high conversion rates and frequent use of small tangential resections [30]. Thirty-five procedures are required to complete the learning curve of RPD with vein resection [30].

Considering that safety is paramount in a new procedure such as RPD, it is important to underscore that mesenteric vessels must be approached carefully to prevent massive bleeding that could be difficult to fix [12]. Although exact figures on incidence of this type of intraoperative complication are missing, emergency conversion due to bleeding was required in 12 of 709 minimally invasive PDs (1.6%), including both RPD and LPD. Multivariable analysis showed that LPD was associated with a higher risk of conversion (OR 5.2, 95% CI. 2.5–10.7; *p* < 0.001) [20]. Despite RPD reduces the risk of massive intraoperative bleeding (versus LPD), we wish to emphasize that surgeons should be prepared to face hemorrhage from major visceral vessels before embarking upon RPD.

The true, and perhaps the only, Achilles heel of RPD is high operative costs [10, 52] that constitute a barrier to implementation of RPD on a larger scale and accumulation of relevant experience at most Institutions. If robotic assistance would come to the same cost of conventional laparoscopy, there would be no rational reason to avoid/limit the use of this technology in patients suitable for minimally invasive PD. A further improvement that would also be needed is availability of dissection instruments specifically suitable for RPD, such as streamlined energy devices with endrowrist® articulation. These instruments are frequently used in either OPD or LPD with good outcomes. To obviate to this lack, some groups still prefer to use a hybrid technique, with sequential laparoscopic dissection and robotic reconstruction, or allow the assistant at the table to use laparoscopic energy devices to enhance robotic dissection.

In conclusion, RPD allows safe implementation of minimally invasive PD. Current results show that RPD is at least equivalent to OPD and LPD, with superiority becoming evident for selected outcome measures. When more centers will have gained enough experience, it is likely that RPD will show clear superiority over alternative approaches.

## Data Availability

This state-of-the-art manuscript is based on data derived from cited articles.
